# Modulation the crosstalk between tumor-associated macrophages and non-small cell lung cancer to inhibit tumor migration and invasion by ginsenoside Rh2

**DOI:** 10.1186/s12885-018-4299-4

**Published:** 2018-05-22

**Authors:** Honglin Li, Nan Huang, Weikang Zhu, Jianchun Wu, Xiaohui Yang, Wenjing Teng, Jianhui Tian, Zhihong Fang, Yingbin Luo, Min Chen, Yan Li

**Affiliations:** 1 Department of Oncology, Shanghai Municipal Hospital of Traditional Chinese Medicine, Shanghai University of Traditional Chinese Medicine, No.274, Zhijiang Road, Jing’an District, Shanghai, 200071 China; 2 Central Laboratory, Tenth People’s Hospital of Tongji University, Shanghai, 200072 China; 3 Department of Oncology, Longhua Hospital, Shanghai University of Traditional Chinese Medicine, Shanghai, 200032 China

**Keywords:** Ginsenoside Rh2 (G-Rh2), Tumor-associated macrophage (TAM), Vascular endothelial growth factor (VEGF), Matrix metalloproteinases (MMPs), Non-small cell lung cancer (NSCLC)

## Abstract

**Background:**

Tumor-associated macrophages (TAMs) play a critical role in modulating the tumor microenvironment and promote tumor metastases. Our studies have demonstrated that ginsenoside Rh2 (G-Rh2), a monomeric compound extracted from ginseng, is a promising anti-tumor agent in lung cancer cells. However, it remains unclear whetherG-Rh2 can modulate the differentiation of TAMs and its interaction with tumor microenvironment. In this study, we investigated how G-Rh2 regulates the phenotype of macrophages and affects the migration of non-small cell lung cancer (NSCLC) cells.

**Methods:**

Murine macrophage-like RAW264.7 cells and human THP-1 monocyte were differentiated into M1 and M2 subsets of macrophages with different cytokines combination, which were further identified by flow cytometry with specific biomarkers. M2 macrophages were sorted out to co-culture with NSCLC cell lines, A549 and H1299. Wound healing assay was performed to examine the cell migration. Expression levels of matrix metalloproteinases 2 and 9 (MMP-2, − 9) and vascular endothelial growth factor-C (VEGF-C) were measured by RT-qPCR and western blot, and the release of VEGF in the supernatant was measured by a VEGF ELISA kit. Finally, modulation of TAMs phenotype and VEGF expression by G-Rh2 was examined in vivo.

**Results:**

We demonstrated that M2 subset of macrophages alternatively differentiated from RAW264.7 or THP-1cells promote migration of NSCLC cells. Further examinations revealed that NSCLC significantly increased the release of VEGF to the media and elevated the expression levels of VEGF at mRNA and protein levels after being co-cultured with M2 macrophages. Similar alterations in MMP-2 and MMP-9 were observed in NSCLC after being co-cultured. Of note,G-Rh2 had a potential to effectively convert M2 phenotype to M1 subset of macrophages. Importantly, G-Rh2 had a preference to decrease the expression levels of VEGF, MMP2, and MMP9 in co-cultured lung cancer cells, over than those in lung cancer cells without co-culturing. Consistently, G-Rh2 reduced M2 macrophage marker CD206 and VEGF expression levels in vivo.

**Conclusions:**

All of these results suggested that M2 subset macrophages drive lung cancer cells with more aggressive phenotypes. G-Rh2 has a potential to convert TAMs from M2 subset to M1 in the microenvironment and prevents lung cancer cell migration, suggesting the therapeutic effects of G-Rh2onlung cancer.

## Background

Lung cancer is the second cancer diagnosed and the first leading cause of cancer-related death. Among these cases, non-small-cell lung cancer (NSCLC)accounts for 80–85% of the total incidence in the world [1]. Major reasons for a poor prognosis are associated with aggressive phenotypes that result in a preference to metastasis at early stage [2–4]. Despite of recent advances in the treatment for NSCLC, there are growing requirements for innovative therapeutic strategies to decrease the mortality of lung cancer [1, 5, 6].

It is well-known that tumor microenvironment is important for cancer development and metastasis. Macrophages are essential immune cells that play a critical role in carcinogenesis and tumor progression in the tumor microenvironment [7], which can be divided into two subsets: the classical subtype of activated macrophage (M1) and the alternative subtype of activated macrophages (M2) [8]. These tumor-associated macrophages (TAMs) may have potential with anti-tumor (M1) or pro-tumor (M2) functions depending on the cytokine milieu of the tumor microenvironment [9]. Of note, more evidence supports that TAMs with M2 phenotype promote tumor progression through complex autocrine and paracrine pathways which are closely associated with tumor malignant proliferation, invasion, and metastasis [8, 9]. Among these factors, matrix metalloproteinases (MMPs) are known to generate a variety of anti-angiogenic peptides. In addition, M2 phenotype of TAMs can also accumulate fibrin, collagen, degrade extracellular matrix (ECM) and promoting tumor growth and metastasis. Moreover, accumulating evidence suggests that TAMs are responsible for releasing several growth factors, cytokines, chemokines, inflammatory mediators and other molecules [10–12]. Many of these molecules including vascular endothelial growth factor(VEGF), platelet derived growth factor (PDGF) and interleukin-10 (IL-10) are associated with tumor growth, poor prognosis and metastasis, [13]. Among these factors, VEGF is a key mediator of tumor-associated metastasis [13].

G-Rh2, a major bioactive ingredient in ginseng, has been shown to have anti-tumor activities against human hepatoma cells, lung cancer cells, and leukemia cells [14–16]. Many reports have demonstrated that mechanisms underlying G-Rh2 to against cancer mainly via arresting cell cycles at G1 phase and activating apoptosis-related pathways, such as Bcl2 family members and caspase signaling [14–16]. Recently,G-Rh2 is reported to inhibit lung cancer cell growth by blocking the PI3K-Akt signaling pathway [17]. Furthermore, the anti-inflammation function of G-Rh2 has attracted many attentions mainly through regulating a critical inflammatory mediator, NF-kappa B (NF-κB) [18]. However, it remains unclear whether G-Rh2 could modulate the macrophage polarization and alter the communication between macrophages and NSCLC, thereby affecting lung cancer progress.

In the present study, we demonstrated that G-Rh2converts the differentiation of macrophages from M2 to M1 phenotype that results in decreasing the levels of MMPs and VEGF and preventing the metastasis of NSCLC cells. Overall, our findings suggest that G-Rh2 has a potential to improve the tumor microenvironment and emphasize the importance of TAMs in cancer progress. This study provides an important rationale for the development of a novel therapeutic strategy in NSCLC patients through the skewing of TAMs phenotype.

## Methods

### Materials

G-Rh2 was obtained from National Standard Material Center (Beijing, China). Dulbecco’s modified Eagle’s medium (DMEM), fetal bovine serum (FBS), and trypsin were bought from GIBCO/BRL (Grand Island, NY, USA). VEGF-ELISA kit was purchased from R&D Systems (Minneapolis, MN, USA). VEGF antibody was from Santa Cruz Biotechnology (Santa Cruz, CA, USA).MMP9 and MMP2antibodies were purchased from Abcam (Cambridge, UK). The flow cytometry antibodies CD206, CD16/32were purchased from Peprotech (New Jersey, NJ, USA). Lipopolysaccharide (LPS) was from Sigma-Aldrich (St. Louis, MO, USA).Interferon-γ (IFN-γ) and interleukin-4 (IL-4) were produced by BioLegend (San Diego, CA, USA).

### Cell lines

The murine macrophage-like cell line RAW264.7, human lung adenocarcinoma cell lines A549 and H1299, and human THP-1 cells were purchased from Shanghai Institute of Biological Science (Catalogue Number TCM13, TCHu150, TCHu160 and SCSP-648, respectively. Shanghai, China).

### Cell culture and polarization of macrophages

These cells were cultured in DMEM media supplemented with 10% FBS,100 U/mL of penicillin, 100 μg/mL of streptomycin at 37 °C in a humidified atmosphere containing 5% CO2.RAW264.7 and THP-1cells were polarized into M1 and M2 macrophages with different stimulation. Combination LPS (100 ng/mL) and IFN-γ(20 ng/mL) were used to generate M1 subset macrophages. IL-4 (20 ng/mL) was used to differentiate cells into M2 subset macrophages.

### Co-culture method

Transwell plate from Corning (NY, USA) with a pore size 0.4 μM was used as a co-culture system. RAW264.7 (5 × 10^5^/mL) or THP-1 (1.5 × 10^5^/mL) were loaded on the upper chamber. Cells were treated with IL-4 (20 ng/mL) for 48 h to differentiate into M2 macrophages. Lung cancer cells A549 or H1299 (2.5 × 10^5^/mL) were loaded in the lower chamber for 24 h. Then, M2 macrophages and lung cancer cells were co-cultured under conditions without serum for 24 h to generate co-cultured lung cancer cells, using lung cancer cells without co-cultured as control. These cells were used for further experiments to be treated with G-Rh2.

### Flow cytometry

After 48 h stimulation, differentiated cells were harvested and identified by flow cytometry with specific makers i.e. CD16/32 for M1 and CD206 for M2 macrophages. M2 macrophages were sorted out using flow cytometry with CD206 marker for further co-culture experiment.

### Cell proliferation assay

In brief, A549, H1299 cells, and respective co-cultured cells were seeded in 96-well plates (3 × 10^3^ cells/well) at the logarithmic phase. After 24 h, cells were treated with different concentrations of G-Rh2 (5, 10, 20, 40, 60, 80, 100, 120 μM) for 72 h. Then, the proliferation of the cells was determined by CCK-8 assay according to manufacturer’s instruction.

### Wound healing assay

The cells were seeded in a 12-well plate to form a monolayer one day before the assay. After making a uniform straight scratch with a pipette tip, cells were incubated for 24 h. Cell motility was assessed by measuring the speed of wound closure at intervals. Each experiment was performed in triplicate.

### Enzyme-linked immunosorbent assay (ELISA)

The concentration of VEGF in the supernatant was determined by ELISA Kit (R&D System). Samples from each group were collected in sterile tubes and centrifuged at 1500 rpm for 15 min to obtain supernatants. The supernatants were analyzed according to the manufacturer’s instructions. Results were presented as picograms of VEGF per milliliter.

### Western blot analysis

Briefly, cells were washed twice with ice-cold phosphate buffer saline(PBS) after treatment with G-Rh2 for 24 h. Next, cells were harvested with ice-cold lysis buffer. Then, cell lysates were centrifuged at 12,000×g for 10 min at 4 °C and collected the supernatant. The total of 50 μg protein per sample was separated by electrophoresis on 8 to 10% SDS-PAGE gel. Then, protein was transferred onto a nitrocellulose membrane. The membrane was blocked with 5% non-fat dry milk for 1 h and incubated with MMP2, MMP9, and VEGF-C (1:1000) primary antibodies overnight at 4 °C. β-actin was used as a loading control.

### Quantitative real-time reverse transcription-PCR

Total RNA isolated from cells using an RNeasy Micro kit (Qiagen) was converted to first-strand cDNA using a high-capacity cDNA reverse transcription kit (Applied Biosystem). Quantitative real-time PCR assays were performed with SYBR Green PCR Master Mix (Applied Biosystems) and a 7900HT Fast Real-time PCR System (Applied Biosystems). All primers were synthesized in Huada Biotechnology Corporation (Shenzhen, China). The sequence of primers was shown in the Table 1. All data were normalized by β-actin.

**Table 1:** RT-qPCR primers used in the study

Gene name	sequence
β-actin	5’-CTGGAACGGTGAAGGTGACA-3’
	5’-AAGGGACTTCCTGTAACAACGCA-3’
MMP2	5’-GCTGGAGACAAATTCTGGAGATACA-3’
	5’-GTATCGAAGGCAGTGGAGAGGA-3’
MMP9	5’-GTATCGAAGGCAGTGGAGAGGA-3’
	5’-CAGGGACAGTTGCTTCTGGA −3’
VEGF	5’-CAGGGACAGTTGCTTCTGGA − 3’
	5’-CAGGGACAGTTGCTTCTGGA − 3’
TNFα	5’-CCCCAAAGGGATGAGAAGTT-3’
	5’-CACTTGGTGGTTTGCTACGA − 3′
iNOS	5′-GTTCTCAGCCCAACAATACAAGA-3′
	5′-GTGGACGGGTCGATGTCAC-3′
ARG-1	5′-CAGAAGAATGGAAGAGTCAG-3′
	5′-CAGAI’ATGCAGGGAGTCACC-3′

### Immunohistochemistry

It was performed as previously described [11]. Briefly, paraffin-embedded tumor samples were cut into 4 μm-thick sections and mounted on polylysine-coated slides. Samples were dewaxed in xylene and rehydrated using a graded series of ethanol solutions. After deparaffinization, endogenous peroxidase activity was blocked by incubation with 3% peroxide-methanol solution at room temperature (RT) for 10 min, and then antigen retrieval was performed at 100 °C in an autoclave for 7 min. After washing with PBS, sections were incubated with primary antibodies against theCD206 monoclonal antibody (clone 10D6, Zhongshan Goldenbridge Biotechnology Co., LTD., Beijing, China) and VEGF-C (Santa Cruz Biotechnology, Santa Cruz, CA, USA)overnight at 4 °C. Next, sections were incubated with aDAKO EnVision kit (DAKO, Glostrup, Denmark) following the manufacturer’s instructions. Finally, sections were faintly counter-stained with hematoxylin and mounted with glycerol gelatin.

### Animal experiments

Female 5-week-old C57BL/6 mice (*n* = 14) were purchased from Shanghai Silaike Experiment Animal Co., Ltd. (Shanghai, China). Animal experiments were conducted in animal room with Specific Pathogen Free (SPF) standards. All animal experiment protocols were approved by Institutional Animal Care and Use Committee. Each mouse was subcutaneously injected 5 × 10^5^ murine lewis lung carcinoma (LLC) cells on right should blade. Then, mice were randomly divided into two groups: vehicle control (*n* = 7) and G-Rh2 (n = 7) which was administered i.p. at 40 mg kg^− 1^ daily for 21 days. Tumor size was measured daily. Then mice were sacrificed after CO_2_ anesthesia. Tumor tissues were isolated and fixed in formalin immediately for further immunohistochemistry experiments.

### Statistical analysis

Statistical analysis was performed using the SPSS statistical package (version 13.0; SPSS Inc., Chicago, IL, USA). All of the data from the quantitative assays are expressed as means ± standard deviation. The significant differences between the groups were evaluated by one-way analysis of variance (ANOVA) and χ2 test. Results were considered statistically significant if the *P* value was less than 0.05.

## Results

### Cells polarization into M2 macrophage

**Fig. 1:**
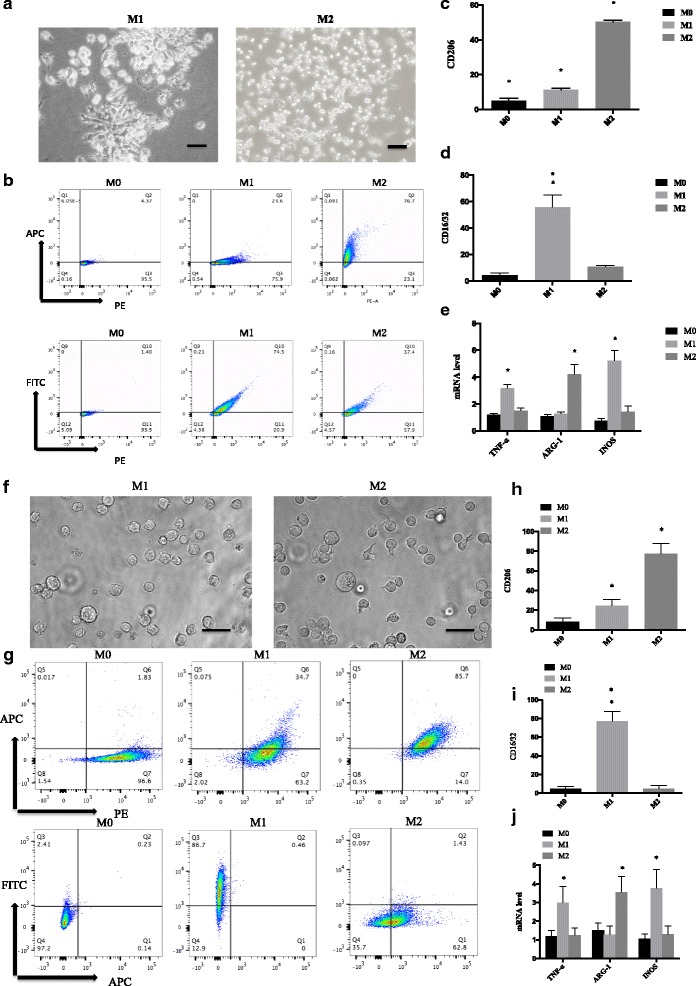
RAW264.7 cells polarization into M2 macrophage. **a** Morphology of the polarized RAW264.7 cells to M1or M2 subsets.RAW264.7 cells were treated with LPS (100 ng/mL) plus INF-γ (20 ng/mL) for 48 h to differentiate into M1. RAW264.7 cells were treated withIL-4 (20 ng/mL) for 48 h to differentiate into M2. The scale bars indicate 200 μM. **b** Identification of the macrophages derived from RAW264.7 cells with specific markers FITC CD16/32 and APC CD206 by FACS. **c** Quantitation of CD206positive cells derived from RAW264.7 cells after different combination treatment for 48 h.**P < 0.01, compared with M0. **d** Quantitation of CD16/32 positive cells derived from RAW264.7 cells after different combination treatment for 48 h. **P < 0.01, compared with M0. **e** RNA was extracted from M1 and M2 macrophages differentiated from RAW264.7 cells. RT-PCR was used to quantitate TNFα, ARG-1, and INOS. * *P* < 0.05, compared with M0 control. **f** Morphology of the polarized THP-1 cells to M1 or M2 subsets. THP-1 cells were treated with LPS (100 ng/mL) plus INF-γ (20 ng/mL) for 48 h to differentiate into M1. THP-1 cells were treated with IL-4 (20 ng/mL) for 48 h to differentiate into M2. The scale bars indicate 200 μM. **g** Identification of the macrophages derived from THP-1 with specific markers FITC CD16/32 and APC CD206 by FACS. **h** Quantitation of CD206 positive cells differentiated from THP-1 cells after different combination treatment for 48 h. *P < 0.05, compared with M0. **i** Quantitation of CD16/32 positive cells differentiated from THP-1 cells after different combination treatment for 48 h. **P < 0.01, compared with M0. **j** RNA was extracted from M1 and M2 macrophages differentiated from THP-1 cells. RT-PCR was used to quantitate TNFα, ARG-1, and INOS. * *P* < 0.05, compared with M0 control

M2 macrophages are considered as an important subtype of TAMs to affect tumor metastasis [19, 20]. In order to investigate how G-Rh2 affects the function of M2 macrophage, unstimulated RAW264.7 cells (M0) were classically treated with LPS (100 ng/mL) and INF-γ(20 ng/mL) for 48 h and differentiated into M1 subset (Fig. 1a) whereas cells stimulated withIL-4 (20 ng/mL) promoted M2macrophage polarization, exhibiting different cellular morphologies between two subsets of macrophages (Fig. 1a). These cells were further identified with specific markers throughflow cytometry analysis. CD206 is a crucial marker for M2 macrophages which was dramatically upregulated after induction by cytokines (Fig. 1b and c). In contrast, markers specific for M1 subtype CD16 and CD32 were remarkably decreased in M2 subtype (Fig. 1b and d). Further examination to detect other biomarkers demonstrated that tumor necrosis factor alpha (TNF-α) and inducible nitric oxide synthase (iNOS) were significantly upregulated in M1 macrophages, whereas arginase 1(ARG-1) was remarkably elevated in M2 subtype (Fig. 1e). To confirm the cell polarization, human THP-1 monocyte was treated with the same combination cytokines as above. M1 and M2 macrophages had different morphologies (Fig. 1f). And M2 subtype displayed higher levels of CD206 (Fig. 1g and h), whereas M1 macrophages had higher levels of CD16/32 than that in M2 subtype (Fig. 1g and i). The expression pattern of TNF-α, iNOS, and ARG-1 in THP-1 derived M1 and M2 macrophages was similar to that derived from RAW264.7 cells (Fig. 1j and e). All of these results suggest that combination of these inflammatory factors is an effective way to polarize M1 and M2 subtypes of macrophage.

### G-Rh2 inhibits the proliferation and migration of lung cancer cells

**Fig. 2:**
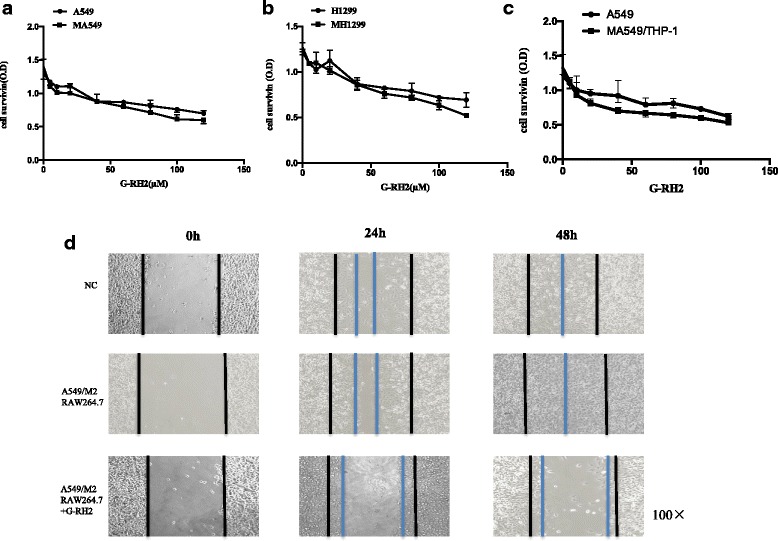
G-Rh2 inhibited the growth and migration of human lung cancer cells. **a** and **b** A549, H1299, andA549/H1299 cells co-cultured with RAW264.7 derived M2 macrophages (3 × 10^3^/well) were treated with different concentrations (5, 10, 20, 40, 60, 80, 100 and 120 μM) of G-Rh2 for 72 h. Cell viability was estimated using CCK-8 assay. Experiments were repeated 3–5 times with the similar results. **c** A549 and A549 co-cultured with THP-1 derived M2 macrophages were treated with different concentrations (5, 10, 20, 40, 60, 80, 100 and 120 μM) of G-Rh2 for 72 h. Cell viability was estimated using CCK-8 assay. Experiments were repeated 3–5 times with the similar results. **d** A549 orco-cultured A549 cells were seeded in a 12-well plate to form a monolayer one day before the assay, and were scratched with a micropipette tip. After washed with PBS, the cells were treated with G-Rh2 (100 μM). The black lines indicate the wound edge; the blue lines indicate the recovery edge. Images for different times (0 h, 24 h, and 48 h) or the negative control (NC) are presented

To mimic the original tumor microenvironment as much as possible, two co-cultured NSCLC cell lines were developed by co-culturing A549 and H1299 with RAW264.7 derived M2 macrophages being alternatively induced, which was named as MA549 and MH1299 cells. A549 and H1299 cells, as well asMA549/MH1299 cells were treated with different concentrations of G-Rh2 for 72 h. As shown in the Fig. 2a and b, g-Rh2had a potential to suppress the growth of A549, H1299 and MA549/MH1299 cells in a dose-dependent manner. It indicated a trend that G-Rh2 at the high doses over 100 μM could inhibit more MA549/MH1299 cell growth than that of not co-cultured cells, but without statistical significance (Fig. 2a and b). In line with above results, A549 cells were co-cultured with THP-1 derived M2 macrophages. G-Rh2 inhibited more co-cultured cell growth than that of A549 cells, but without significant difference (Fig. 2c). To further study the inhibitory effects of G-Rh2on cell migration, we performed a scratch wound model in the presence of mitomycin C to inhibit cell proliferation. Compared with negative control cells (NC), Co-cultured A549cells migrated faster at two time-points of 24 and 48 h, indicating the mobility of NSCLC cells after co-culture was intensively increased (Fig. 2d). Aftertreatment with G-Rh2 (100 μM), the mobility of co-cultured A549 cells was effectively blocked after 24 h (Fig. 2d). Furthermore, cells almost lost mobility after48 hours exposure to G-Rh2 (Fig. 2d). This finding indicates that G-Rh2 is a potent compound to prevent the migration of co-cultured NSCLC cells.

### G-Rh2 reverses the phenotype of M2 macrophages to M1 subtype

**Fig. 3:**
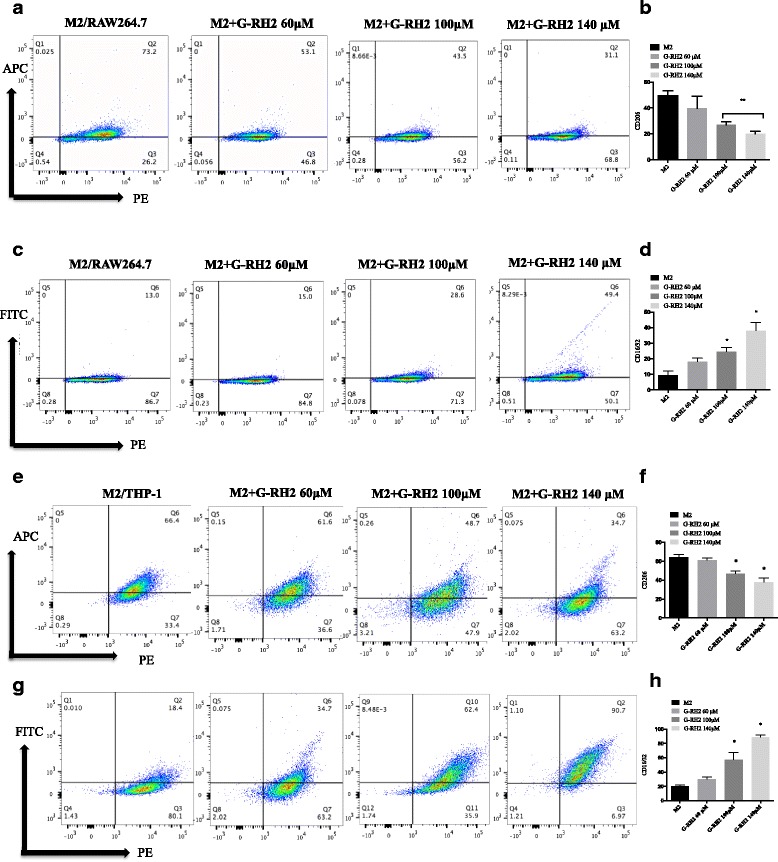
G-Rh2 reversed the phenotype of M2 macrophage into M1 subset. **a** and **b** M2 macrophages derived from RAW264.7 were treated with different concentrations of G-Rh2 for 24 h. These treated cells were harvested and analyzed through flow cytometry with CD206 marker. **c** and **d** M2 macrophages derived from RAW264.7were treated with different concentrations of G-Rh2 for 24 h. These treated cells were harvested and analyzed through flow cytometry with M1 marker CD16/32.The indicated differences are significant: * *P* < 0.05, **P < 0.01. **e** and **f** M2 macrophages derived from THP-1 were treated with different concentrations of G-Rh2 for 24 h. These treated cells were harvested and analyzed through flow cytometry with CD206 marker. **g** and **h** M2 macrophages derived from THP-1 were treated with different concentrations of G-Rh2 for 24 h. These treated cells were harvested and analyzed through flow cytometry with M1 marker CD16/32. The indicated differences are significant: * *P* < 0.05

Since the interaction between TAMs and cancer cells is the key factor to promote cancer metastasis [21], a question was raised concerning whether G-Rh2 affects the phenotype of M2 TAMs. M2 macrophages were identified and sorted out using flow cytometry by detection of the CD206 marker. Then, sorted M2 cells were treated with different doses of G-Rh2 for 24 h. These treated cells were harvested and analyzed through flow cytometry with characteristic markers. As shown in Fig. 3, G-Rh2 significantly reduced the expression of CD206expression in M2 macrophages derived from RAW264.7 in a dose-dependent manner (Fig. 3a and b). It was particularly interesting to detect that M1 markers CD16/32 expression was simultaneously increased in a dose-responsive way (Fig. 3c and d) after G-Rh2 treatment. To further confirm this function of G-Rh2,M2 macrophages differentiated from human THP-1 cells were treated with different concentrations of G-Rh2 for 24 h. The similar subtype switch was observed that M1 markers CD16/32 was increased whereas M2 phenotype CD206 was decreased (Fig. 3e-h). All of these results indicate that G-Rh2 has a potential to shift M2 phenotype to M1 thereby affecting the biological function of TAMs.

### G-Rh2 decreases the secretion and the mRNA levels of VEGF-C, MMP2, and MMP9 in co-cultured lung cancer cells

**Fig. 4:**
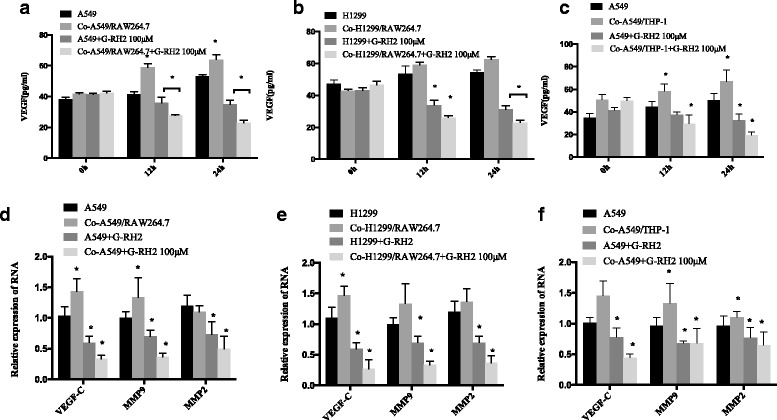
G-Rh2 decreased the release and mRNA levels of VEGF and MMPs by lung cancer cells. **a** and **b** A549/H1299 and co-cultured with M2 derived from RAW264.7 cells were treated with G-Rh2(100 μM)for different time points as indicated. Supernatants were harvested and VEGF levels were measured by an ELISA kit. Experiments were repeated at least three times. *P < 0.05compared with the control. **c** A549 and co-cultured with M2 derived from THP-1 cells were treated with G-Rh2 (100 μM) for different time points as indicated. Supernatants were harvested and VEGF levels were measured by an ELISA kit. Experiments were repeated at least three times. * *P* < 0.05 compared with the control. **d** and **e** A549, Co-A549, H1299 and Co-H1299 cells were treated with different concentrations of G-Rh2 for 24 h. Cells were harvested for RNA extraction. The mRNA expression levels of VEGF-C, MMP9 and MMP2 were quantified by RT-PCR. * *P* < 0.05 compared with control. **f** A549 and co-cultured with M2 derived from THP-1 cells were treated with different concentrations of G-Rh2 for 24 h. Cells were harvested for RNA extraction. The mRNA expression levels of VEGF-C, MMP9 and MMP2 were quantified by RT-PCR. * *P* < 0.05 compared with control

Compelling evidence indicates that VEGF and MMPs are important factors involving in the cancer metastases, which may be regulated by M2 macrophages in tumor microenvironment [22–24]. The secretory levels of VEGF-C were measured by ELISA. Results showed that VEGF levels were significantly increased in co-cultured A549 cells after being co-cultured 12 and 24 h with M2 macrophages derived from RAW264.7, compared with that in the media of A549 cells (Fig. 4a). G-Rh2 reduced the basal levels of VEGF in A549 culture media and decreased more in co-culturing system (Fig. 4a). As for another NSCLC cell line H1299, there was a tendency to upregulate VEGF-C levels after co-cultured with M2 macrophages derived from RAW264.7, but without significant difference (Fig. 4b). Similarly as in A549 or co-cultured A549, G-Rh2 remarkably inhibited the secretion of VEGF in H1299 and co-cultured H1299, especially in co-culturing system (Fig. 4b). When A549 cells were co-cultured with M2 macrophages differentiated from THP-1 cells, secretion of VEGF-C was increased and G-Rh2 remarkably inhibited the up-regulation of VEGF-C (Fig. 4c). In agreement with the secretory levels of VEGF, VEGF-C mRNA expression levels were increased in Co-A549 and Co-H1299 cells, compared to their respective controls. G-Rh2 significantly reduced the mRNA levels of VEGF-C in A549 and H1299 cells and effectively blocked the induction of VEGF by co-culturing with M2 macrophages derived from RAW264.7 or THP-1 (Fig. 4d-f). Similar regulatory patterns were observed in the expression of MMP9 and MMP2 mRNA by G-Rh2 in two lung cancer cell lines with or without being co-cultured (Fig. 4d-f). These observations suggest that M2 macrophages promote the expression of VEGF and MMPs in lung cancer cells.

### G-Rh2 significantly reduces the protein levels of VEGF-C, MMP2, and MMP9 in co-cultured lung cancer cells

**Fig. 5:**
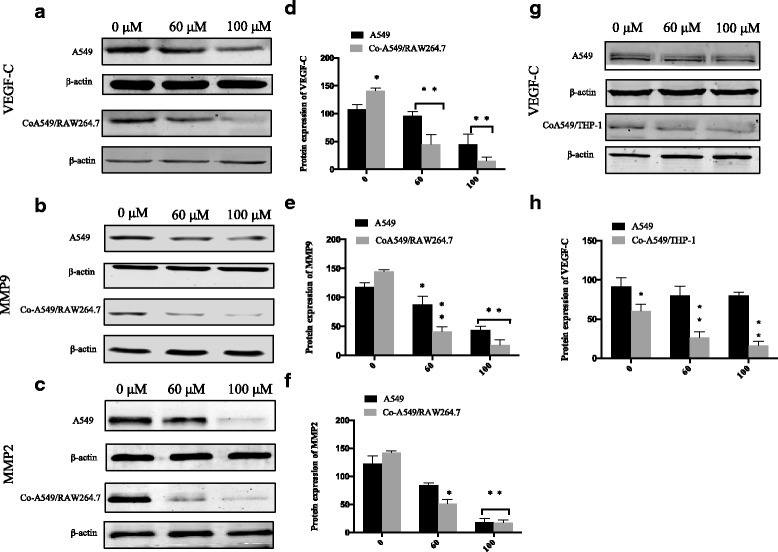
G-Rh2 downregulated protein expression levels of VEGF-C, MMP9 and MMP2 in NSCLC cells. **a-c** A549 and co-cultured A549 with RAW264.7 derived M2 cells were treated with different concentrations of G-Rh2 for 24 h. Cell lysates were harvested. Protein expression levels of VEGF-C, MMP9 and MMP2 were examined by western blot. β-actin was used as a loading control. **d**-**f**)Quantification of VEGF-C, MMP9, and MMP-2 bands through quantification software. * *P* < 0.05, **P < 0.01, compared with respective control. **g** A549 and co-cultured A549 with THP-1 derived M2 cells were treated with different concentrations of G-Rh2 for 24 h. Cell lysates were harvested. Protein expression levels of VEGF-C was examined by western blot. β-actin was used as a loading control. **h** Quantification of VEGF-C bands through quantification software. * *P* < 0.05, **P < 0.01, compared with respective control

Since VEGF and MMPs promote cancer cell invasion and metastasis mainly through their respective proteins [24–26], thereby protein expression levels were measured by Western blotting. In line with the regulation of mRNA expression, the protein expression levels of VEGF-C were decreased by G-Rh2 at high concentration of 100 μM in A549 cells, whereas G-Rh2 started to decrease VEGF-C at concentration of 60 μM in co-cultured A549 with M2 derived from RAW264.7 cells. Given the same concentration of G-Rh2 at 100 μM, it was more potent to reduce VEGF-C protein levels in Co-A549 cells over than that in A549 cells without being co-cultured with M2 macrophages (Fig. 5a). As for theMMP9, G-Rh2 weakly reduced the protein levels in A549 even at high concentration (Fig. 5b). In contrast, G-Rh2 clearly decreased MMP9 protein expression at 60μMin co-cultured A549 cells (Fig. 5b). Similarly, G-Rh2 significantly blocked the MMP2 protein levels at high concentration in A549 cells, while MMP2 protein levels were remarkable reduced at low concentration in co-cultured A549 cells (Fig. 5c). The quantification results were consistent with that from immunoblotting (Fig. 5c-f). In another co-culturing system that A549 with M2 differentiated from THP-1 cells, VEGF protein levels was weakly reduced by G-Rh2 at high concentration (100 μM) in A549 cells, but G-Rh2 remarkably decreased VEGF protein levels at low concentration of 60 μM (Fig. 5g). Interestingly, total VEGF levels in co-cultured A549 with M2 derived from THP-1 cells were lower than that of A549 cells (Fig. 5h). This was different from that in co-cultured A549 with M2 derived from RAW264.7 cells expressing higher levels of VEGF than A549 cells (Fig. 5d). Our results demonstrated that M2 macrophages modulate the biological behaviors of lung cancer cells and G-Rh2 displays a special preference to block expression of these aggressive factors related with cancer malignancy under co-cultured conditions.

### G-Rh2 decreases VEGF-C and CD206expression in vivo

**Fig. 6:**
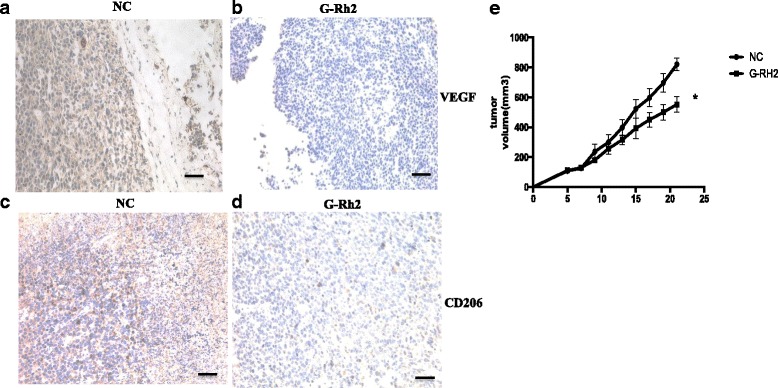
G-Rh2 decreased VEGF-C and CD206 expression in vivo. **a-d** Female 5-week-old C57 mice (*n* = 14) were subcutaneously injected with murine lung cancer cells. Then, they were randomly divided into two groups i.e. vehicle control and G-Rh2 treated groups. After 21 days treatment, tumors were taken out for immunohistochemical staining with VEGF-C and CD206. **e** Tumor size was measured daily. G-Rh2 administration reduced the tumor size. * *P* < 0.05 compared with control group

To confirm the regulatory function of G-Rh2 in the communication between TAMs and lung cancer cells, murine lewis lung carcinoma cells (LLC) were injected subcutaneously in C57BL/6 mice. Then, mice were divided into two groups i.e. vehicle control and G-Rh2 administered group. Tumor size was measured daily. After 21 days, mice were sacrificed and tumor tissues were fixed for immunohistochemistry. Strong cytoplasmic staining of VEGF-C was predominantly observed in cancer cells and tumor stromal cells (Fig. 6a) and G-Rh2 significantly inhibited VEGF-C expression (Fig. 6b). As for the marker of M2 macrophages, CD206 was highly expressed on the cell membrane and cytoplasm in the infiltrative macrophages among the tumor cells (Fig. 6c). G-Rh2 remarkably decreased CD206 expression (Fig. 6d). Importantly, G-Rh2 also significantly reduced the tumor size compared with vehicle control group (Fig. 6e). These results clued that G-Rh2 can prevent macrophages from differentiation into the M2 subtype, which might disassociate the communication between TAMs and lung cancer cells.

## Discussion

The complex communication between tumor cells and TAMs within the tumor microenvironment affects the cancer development [2, 27]. TAMs can be either pro- or anti-tumorigenic in response to different environmental cues [2, 28, 29]. Thus, how to polarization macrophages towards therapeutic effects is a desired strategy for cancer treatment. Our findings demonstrate that M2 subset of macrophages are potent to increase migration and upregulate expression of angiogenesis and invasion associated factors such as VEGF and MMPs in lung cancer cells. Importantly, G-Rh2 significantly induces M2 macrophage differentiation into the M1 phenotype which leads to the prevention of migration and less expression of these angiogenetic factors by lung cancer cells. All of these suggest that G-Rh2 is a therapeutic candidate to improve the microenvironment of lung cancer.

Growing evidence has shown that G-Rh2 activates apoptosis-related signal pathways to inhibit cancer cell growth [14–16]. In agreement with those results, we also observe that G-Rh2 significantly inhibits lung cancer cell growth in vitro and in vivo. Importantly, we provide a novel mechanistic finding that G-Rh2 has a potential to inhibit invasion and migration of lung cancer cells via modulation the phenotypes of macrophages. Our results indicate that alternative differentiation of the M2 phenotype of macrophage into the M1 subset by G-Rh2 benefits the therapy for lung cancer. Nevertheless, it is still unclear how G-Rh2 affects the polarization of macrophages. Xie et al. reported that G-Rh2 can inhibit the PI3K/Akt signal pathway [17], which might be a candidate signal being involved in the regulation of macrophage differentiation [30]. Of note, macrophages display remarkable plasticity and can change their physiology in response to environmental changes. These alterations can give rise to different populations of cells with distinct functions [31, 32].

Functionally, macrophages are broadly classified into two groups, proinflammatoryM1 and anti-inflammatoryM2 according to the secreted cytokines [31–33]. Interestingly, M1 macrophages have anti-tumor activities, whereas M2 subset exhibits pro-tumorigenic features [31–33]. These distinct functions of M1 and M2 macrophages in inflammation and cancer provide an important rationale for the clinic to generate a personized macrophages differentiation strategy according to different diseases [34–36]. However, it should be pointed out here that differentiation of macrophages is a complicated processing with multiple growth factors and cytokines secreted by macrophages and cancer cells [31–33]. Among these factors, VEGF is a key angiogenic factor secreted by tumors, as well as by macrophages in the tumor microenvironment [33] which has been confirmed to be associated with poor prognosis for cancer patients [12, 26, 37]. Moreover, the distribution of TAMs is affected by these angiogenic factors. Despite of the fact that TAMs widely distribute in the tumor microenvironment including the invasive tumor edge, center of tumor mass, and perivascular areas [20],the enrichment of perivascular macrophages has been shown to correlate with increased tumor angiogenesis, distant metastasis, and poor prognosis [20, 38–40]. Consistent with these findings, our results demonstrate that M2 macrophages significantly upregulate expression levels of angiogenesis-related molecules such as VEGF, MMP2, and MMP9 after being co-cultured with lung cancer cells, resulting in the poor prognosis of lung cancer [41, 42]. A clinical relevant finding in the present study is that G-Rh2 has a potential to remarkably downregulate the expression of these factors.

## Conclusions

Our results suggest that M2 subset of macrophages are potent to increase migration and upregulate expression of angiogenesis and invasion associated factors such as VEGF and MMPs after being co-cultured with lung cancer cells. Importantly, G-Rh2 can significantly induce M2 macrophage differentiation into M1 phenotype which leads to the prevention of migration and less expression of these angiogenetic factors by lung cancer cells. All of these results suggest that G-Rh2 can improve the tumor environment through modulating phenotype of TAMs in lung cancer.

## Abbreviations

Co-A549: co-cultured A549 cells
Co-H1299: co-cultured H1299 cells
ECM: extracellular matrix
ELISA: enzyme-linked immunosorbent assay
FACS: fluorescence activated cell sorter
G-Rh2: Ginsenoside Rh2
IL-10: interleukin-10
MMPs: matrix metalloproteinases
NC: negative control
NSCLC: non-small cell lung cancer
PDGF: platelet derived growth factor
RT-qPCR: reverse transcription-quantitative real-time PCR
TAMs: tumor-associated macrophages
VEGF: vascular endothelial growth factor
